# Notification of bacterial strains made available by the UK National Collection of Type Cultures in 2023

**DOI:** 10.1099/acmi.0.001015.v3

**Published:** 2025-10-22

**Authors:** Jake David Turnbull, Jo Dicks, Rachael Adkin, Alexander Dickinson, Dorota Kaushal, Mojisola Semowo, The NCTC 2023 Depositors Cohort, Hannah McGregor

**Affiliations:** 1 The National Collection of Type Cultures, UK Health Security Agency, 61 Colindale Avenue, Colindale, London, UK, NW9 5EQ; 2 Author group listed below

**Keywords:** bacteria, culture collections, informatics, novel taxa, pathogen

## Abstract

Many microbial culture collections, like the UK’s National Collection of Type Cultures, add biological material such as bacterial strains to their holdings over time in a process known as accessioning. Here, we report on the 101 bacterial strains made available to scientists in the UK and globally by the National Collection of Type Cultures in 2023. Strains that are received are preserved, identified to species level and confirmed to be viable and pure. Genomic and metadata (where these are available) are made accessible via the UKHSA Culture Collections online catalogue. Commentary on the strains’ provenance and significance is presented, and wider trends in accessioning between 2017 and 2023 are examined. On average, ~101 strains were made available to the scientific community each year between 2017 and 2023. Fewer strains of veterinary provenance were made available than any other kind of strain, highlighting a need to accession more of these strains. However, there has been growth in the proportion of strains that are either antimicrobial resistant or type strains of novel microbial taxa, demonstrating that the NCTC program of accessioning helps support its function as a contemporary public health resource and repository for prokaryotic taxonomists.

## Data Summary

The assembled set of 101 strains described below was first made available from the UK’s National Collection of Type Cultures in 2023 to the global scientific community, alongside the following data that are accessible via this manuscript and the UKHSA Culture Collection online catalogue:

(a) Strain metadata, including from what, where and when strains were isolated, where these data are available in Table S1.

(b) Collated whole-genome sequence data for strains obtained from peer-reviewed manuscripts where these data are available.

(c) Certificates of analysis for each strain detailing the outcome of microbiological testing, the contents of which are summarized in Table S2.

The full details for each strain (citations for the full taxon descriptions, strain provenance, validated growth conditions, a selected bibliography for each strain where relevant etc.) can be obtained from the NCTC online catalogue; https://www.culturecollections.org.uk/products/bacteria-and-mycoplasmas. Links to the catalogue entry for each strain can be found in Table 1 in the Data S1, as can the links to the requisite genomic data wherever it is available. Confirmation of strain purity, viability and species-level identification is captured in the certificates of analysis provided with each bacterial strain obtained from the NCTC.

## Significance as a bioresource to the community

The National Collection of Type Cultures (NCTC) is an accessible collection of bacterial strains located in the UK. Strains are continually added (accessioned) to the collection each year, which allows the NCTC to meet the needs of the scientific community by providing contemporaneous biological material, e.g. recently described bacterial species or bacterial strains with uncommon, novel or emergent traits of interest. Where possible, bacterial strains are provided with matched whole-genome sequence data and comprehensive metadata. The NCTC also serves as a centralized resource from which bacterial strains with authenticated phenotypic or genomic traits can be obtained, allowing for more robust reproducibility in microbiological testing at large. It also serves as an archive of biological material, allowing for irreplaceable biological material (such as the Murray Collection of pre-antibiotic era *Enterobacteriaceae*) to be preserved and maintained and not become ‘lost to science’.

## Introduction

Repositories and collections of biological materials are utilized frequently in scientific investigation. The variety of such collections are innumerable in their scope, holdings, use, management and purpose and can range from the contents of a laboratory freezer held privately by a university-based researcher to nationally distributed infrastructure that is designed to supply biological material to the scientific community. The UK National Collection of Type Cultures (NCTC) is one such of the latterly described collections. It is currently composed of ~6,000 bacterial strains of medical and veterinary relevance and is hosted by the UK Health Security Agency (UKHSA).

As with many biological collections, material is acquired and added to the NCTC over time (accessioning). Unlike some biological collections, the strains are then made accessible to science and industry through UK and worldwide distribution for use as reference materials in research and microbiological testing. Befitting the NCTC’s role as a public health resource, many strains are provided for use as controls in diagnostic testing [1], in research related to the understanding of infectious disease and in the improvement of human health.

Approximately 1,000 of the strains held by the NCTC are taxonomic type strains, which are the bacterial strains on which the descriptions of microbial taxa are based. These are typically the first strain of a taxon to be isolated and are frequently denoted by ^T^, e.g. NCTC 11184^T^. The International Code of Nomenclature of Prokaryotes (ICNP) [2] provides a structured and harmonised system by which names are applied to microbial taxa through publication in the *International Journal of Systematic and Evolutionary Microbiology* (*IJSEM*). The ICNP specifies that type strains should be maintained in pure culture in accessible culture collections such as the NCTC, and fulfilling the requirements of the ICNP allows for valid publication of the name of a given microbial taxon [2].

The ICNP also specifies that type strains should be kept in collections hosted by at least two different countries [2]. Microbial cultures stored in any format diminish in viability over time, sometimes to the point where live culture is no longer recoverable. Loss of viability is an infrequent but unfortunate aspect of maintaining any collection of living things, including microbes. Maintaining type strains in multiple collections, especially those that are fastidious or challenging to preserve, highlights the value of culture collections in preserving precious or challenging-to-obtain biological material.

Previously, the NCTC has published lists of the 47 and 125 strains made available to the scientific community in 2021 [3] and 2022 [4], respectively. This itself builds on an irregular practice of reporting and describing accessioned strains in reports to governing entities, laboratory bulletins [5,6] and eight editions of print catalogues published between 1922 and 1994 [7,9].

Here, we present the 101 bacterial strains made available by the National Collection of Type Cultures in 2023, alongside commentary on the strains and a longitudinal description of accessioning trends over time.

## Methods

The methods used to accession bacterial strains into the NCTC have been reported previously in Turnbull *et al*. [4]. In the interests of clarity, key concepts and aspects of the methodology are outlined below.

Requests for the accession of bacterial strains into the NCTC are either received by the NCTC or proactively initiated by NCTC staff. Requests are formalized by review (by NCTC curatorial staff) of a deposit form completed by the depositor.

Following a successful review of the deposit form and receipt of the bacterial strains, the strains are typically preserved by freeze-drying and are checked for viability, purity and conformity to expected species-level identification and selected phenotypic traits.

All strains are Gram-stained, tested for catalase and oxidase production, and tested for motility. All strains are identified to species level using a Bruker MALDI-TOF, except for the proposed type strains of novel species and NCTC 14628. Proposed type strains of novel species are not identifiable to the species level by the Bruker MALDI-TOF using the non-bespoke databases used by the NCTC. Species-level identification of proposed type strains is obtained by returning the freeze-dried strains to the depositor, where they are compared to original/reference stocks. NCTC 14628 was not identifiable using MALDI-TOF and was identified to species level using 16S rRNA gene analysis.

Furthermore, the serotypes of the *Salmonella* strains were determined by the UKHSA Gastrointestinal Bacteria Reference Unit (https://www.gov.uk/guidance/gbru-reference-and-diagnostic-services).

Where third-party genomic data are provided, these are recorded and collated. The third-party genomic data that are presented below are taken from previously published work, typically from published species descriptions [10,20] or examinations of groups of strains [21,22]. These data are not checked routinely here, as they have already been peer-reviewed and are not used to confirm species-level identification for each strain except for NCTC 14628. DNA extraction, whole-genome sequencing (WGS) protocols and WGS assembly can be found within the primary citation of each strain where whole-genome sequence data are available.

Bacterial strains are then integrated into the NCTC online catalogues and inventory systems and are made available to the scientific community via the NCTC online catalogue. Confirmation of strain purity, viability and species-level identification is captured in the certificates of analysis provided with each bacterial strain obtained from the NCTC. The certificates of analysis are accessible via the NCTC online catalogue, and the compiled data obtained from the certificates of analysis are available in Table S2 (available in the online Supplementary Material). The NCTC online catalogue is accessible via the UKHSA Culture Collections website: https://www.culturecollections.org.uk.

The strains are presented below alongside primary references, available genomic data and a designated key attribute. The strains are categorized by attributes consistent with those presented in Turnbull *et al*. [4] and are assigned primarily to facilitate organised discussion of them. Attributes are assigned hierarchically on the basis listed in Flowchart S1 (SF1). When attributes are applied, they are mutually exclusive, which may not necessarily reflect biological reality, e.g. a strain may be both a ‘Contemporary or significant veterinary isolate’ and an ‘Antimicrobial resistant strain’ but assigned to the category ‘Antimicrobial resistant strain’. The discretion of the authors is exercised in these instances.

Additionally, the number and proportions of microbial strains made available between 2017 and 2023 (as grouped by the above attributes) have been visualized, as have the numbers of microbial strains belonging to each taxonomic order with the wider longitudinal trends in NCTC accessioning discussed.

## Results and discussion: bacterial strains made available by the NCTC and an overview of bacterial strains made available by the NCTC between 2017 and 2023

### Bacterial strains made available by the NCTC in 2023

One hundred one bacterial strains submitted from 13 individuals across 10 different institutes were made available to the scientific community by the NCTC in 2023. Three of the ten institutes are based in the UK. All strains are listed in Table 1. Identification data, as they are listed on the certificates of analysis provided with each strain, are available in Table S2.

**Table 1.** The 125 strains made available by the NCTC in 2023, including 11 type strains of novel taxa, 8 type strains described prior to accession with validly published names, 1 veterinary strain, 2 contemporary clinical strains of human provenance, 15 antimicrobial resistant isolates, 3 laboratory reference strains and 61 strains that were obtained from a legacy strain archive (the Murray collection of pre-antibiotic era *Enterobacteriaceae* [22])

NCTC ID	Species name	Primary reference	Strain attribute	Available whole-genome sequence data
NCTC 14451 ^T^	*Staphylococcus canis*	10	Type strain of novel taxon	GCF_016238445.1^a^
NCTC 14562 ^T^	*Microbacterium pullorum*	11	Type strain of novel taxon	GCA_014836535.1^a^
NCTC 14625 ^T^	*Terrisporobacter hibernicus*	12	Type strain of novel taxon	GCF_020809405.1^a^
NCTC 14661 ^T^	*Vibrio floridensis*	13	Type strain of novel taxon	GCF_021852965.1^a^
NCTC 14700 ^T^	*Oerskovia rustica*	11	Type strain of novel taxon	GCA_014836555.1^a^
NCTC 14701 ^T^	*Clostridium gallinarum*	11	Type strain of novel taxon	GCA_014836325.1^a^
NCTC 14703 ^T^	*Sporosarcina quadrami*	11	Type strain of novel taxon	GCA_014836615.1^a^
NCTC 14721 ^T^	*Neisseria montereyensis*	14	Type strain of novel taxon	GCF_024834045.1^a^
NCTC 14723 ^T^	*Staphylococcus marylandisciuri*	15	Type strain of novel taxon	GCF_025519785.1^a^
NCTC 14727 ^T^	*Streptococcus sciuri*	15	Type strain of novel taxon	GCF_024814375.1^a^
NCTC 14628 ^T^	*Legionella drozanskii*	16	Type strain, previously described	GCF_900640075.1^a^
NCTC 14630 ^T^	*Legionella maioricensis*	17	Type strain, previously described	JAJKBJ000000000
NCTC 14681 ^T^	*Neisseria perflava*	18	Type strain, previously described	GCF_023472875.1^a^
NCTC 14684 ^T^	*Dolosigranulum pigrum*	30	Type strain, previously described	–
NCTC 14686 ^T^	*Streptococcus pluranimalium*	28	Type strain, previously described	–
NCTC 14687 ^T^	*Streptococcus ictaluri*	27	Type strain, previously described	–
NCTC 14688 ^T^	*Streptococcus plurextorum*	19	Type strain, previously described	GCF_000423745.1^a^
NCTC 14693 ^T^	*Lacrimispora algidixylanolytica*	20	Type strain, previously described	GCF_003609635.1^a^
NCTC 14574 ^T^	‘Candidatus Phocaeicola gallinarum’	11	Contemporary or significant veterinary isolate	GCA_014837055.1^a^
NCTC 14717	*Erysipelothrix rhusiopathiae*	–	Contemporary or significant veterinary isolate	–
NCTC 14851	*Streptococcus pyogenes*	–	Contemporary or significant human clinical isolate	–
NCTC 14852	*Streptococcus pyogenes*	–	Contemporary or significant human clinical isolate	–
NCTC 14653	*Leclercia adecarboxylata*	–	Antimicrobial resistant	–
NCTC 14660	*Leclercia adecarboxylata*	–	Antimicrobial resistant	–
NCTC 14894	*Streptococcus agalactiae*	21	Antimicrobial resistant	ERR1742051^b^
NCTC 14895	*Streptococcus agalactiae*	21	Antimicrobial resistant	ERR1741366^b^
NCTC 14896	*Streptococcus agalactiae*	21	Antimicrobial resistant	ERR1741677^b^
NCTC 14897	*Streptococcus agalactiae*	21	Antimicrobial resistant	ERR1742116^b^
NCTC 14898	*Streptococcus agalactiae*	21	Antimicrobial resistant	ERR1742139^b^
NCTC 14900	*Streptococcus agalactiae*	21	Antimicrobial resistant	ERR1741407^b^
NCTC 14901	*Streptococcus agalactiae*	21	Antimicrobial resistant	ERR1741573^b^
NCTC 14902	*Streptococcus agalactiae*	21	Antimicrobial resistant	ERR1742140^b^
NCTC 14905	*Streptococcus agalactiae*	21	Antimicrobial resistant	ERR1741823^b^
NCTC 14906	*Streptococcus agalactiae*	21	Antimicrobial resistant	ERR1741535^b^
NCTC 14907	*Streptococcus agalactiae*	21	Antimicrobial resistant	ERR1741765^b^
NCTC 14908	*Streptococcus agalactiae*	21	Antimicrobial resistant	ERR1741835^b^
NCTC 14909	*Streptococcus agalactiae*	21	Antimicrobial resistant	ERR1741614^b^
NCTC 14679	*Clostridium perfringens*	37	Laboratory reference strain	–
NCTC 14692	*Escherichia coli*	–	Laboratory reference strain	–
NCTC 14916	*Escherichia coli*	–	Laboratory reference strain	–
NCTC 14254	*Salmonella enterica* subsp. *enterica* serotype Thompson	22	Obtained from a legacy strain archive	ERS203069^b^
NCTC 14255	*Salmonella enterica* subsp. *enterica* serotype Bispedjerg	22	Obtained from a legacy strain archive	ERS203071^b^
NCTC 14707	*Salmonella enterica* subsp. *enterica* serotype Virchow	22	Obtained from a legacy strain archive	ERS179742^b^
NCTC 14708	*Salmonella enterica* subsp. *enterica* serotype Thompson	22	Obtained from a legacy strain archive	ERS179680^b^
NCTC 14709	*Salmonella enterica* subsp. *enterica* serotype Bareilly	22	Obtained from a legacy strain archive	ERS179688^b^
NCTC 14710	*Salmonella enterica* subsp. *enterica* serotype Anatum	22	Obtained from a legacy strain archive	ERS179686^b^
NCTC 14711	*Salmonella enterica* subsp. *enterica* serotype Enteritidis	22	Obtained from a legacy strain archive	ERS180054^b^
NCTC 14743	*Salmonella enterica* subsp. *enterica* serotype Enteritidis	22	Obtained from a legacy strain archive	ERS222740^b^
NCTC 14744	*Escherichia coli*	22	Obtained from a legacy strain archive	ERS174283^b^
NCTC 14745	*Escherichia coli*	22	Obtained from a legacy strain archive	–
NCTC 14746	*Escherichia coli*	22	Obtained from a legacy strain archive	ERS179675^b^
NCTC 14747	*Salmonella enterica* subsp. *enterica* serotype Bovismorbificans	22	Obtained from a legacy strain archive	ERS179678^b^
NCTC 14748	*Salmonella enterica* subsp. *enterica* serotype Eastbourne	22	Obtained from a legacy strain archive	ERS179718^b^
NCTC 14749	*Salmonella enterica* subsp. *enterica* serotype Hvittingfoss	22	Obtained from a legacy strain archive	ERS179726^b^
NCTC 14750	*Salmonella enterica* subsp. *enterica* serotype London	22	Obtained from a legacy strain archive	ERS179687^b^
NCTC 14751	*Salmonella enterica* subsp. *enterica* serotype Bareilly	22	Obtained from a legacy strain archive	–
NCTC 14752	*Salmonella enterica* subsp. *enterica* serotype Bareilly	22	Obtained from a legacy strain archive	ERS179696^b^
NCTC 14753	*Salmonella enterica* subsp. *enterica* serotype Reading	22	Obtained from a legacy strain archive	ERS179712^b^
NCTC 14754	*Salmonella enterica* subsp. *enterica* serotype Heidelberg	22	Obtained from a legacy strain archive	ERS179710^b^
NCTC 14755	*Salmonella enterica* subsp. *enterica* serotype Poona	22	Obtained from a legacy strain archive	ERS179681^b^
NCTC 14756	*Salmonella enterica* subsp. *enterica* serotype Bispebjerg	22	Obtained from a legacy strain archive	ERS179705^b^
NCTC 14757	*Salmonella enterica* subsp. *enterica* serotype Reading	22	Obtained from a legacy strain archive	ERS179713^b^
NCTC 14758	*Salmonella enterica* subsp. *enterica* serotype Eastbourne	22	Obtained from a legacy strain archive	ERS179721^b^
NCTC 14759	*Salmonella enterica* subsp. *enterica* serotype Oslo	22	Obtained from a legacy strain archive	ERS179737^b^
NCTC 14760	*Salmonella enterica* subsp. *enterica* serotype Stanley	22	Obtained from a legacy strain archive	ERS179745^b^
NCTC 14761	*Salmonella enterica* subsp. *enterica* serotype Stanley	22	Obtained from a legacy strain archive	–
NCTC 14762	*Salmonella enterica* subsp. *enterica* serotype Potsdam	22	Obtained from a legacy strain archive	–
NCTC 14763	*Salmonella enterica* subsp. *enterica* serotype Dublin	22	Obtained from a legacy strain archive	ERS203018^b^
NCTC 14764	*Salmonella enterica* subsp. *enterica* serotype Blegdam	22	Obtained from a legacy strain archive	ERS203019^b^
NCTC 14765	*Salmonella enterica* subsp. *enterica* serotype Heidelberg	22	Obtained from a legacy strain archive	ERS203037^b^
NCTC 14766	*Salmonella enterica* subsp. *enterica* serotype Eastbourne	22	Obtained from a legacy strain archive	–
NCTC 14805	*Escherichia coli*	22	Obtained from a legacy strain archive	–
NCTC 14806	*Salmonella enterica* subsp. *enterica* serotype Abortusovis	22	Obtained from a legacy strain archive	ERS179720^b^
NCTC 14807	*Salmonella enterica* subsp. *enterica* serotype Abortusovis	22	Obtained from a legacy strain archive	ERS179728^b^
NCTC 14808	*Salmonella enterica* subsp. *enterica* serotype Potsdam	22	Obtained from a legacy strain archive	ERS179673^b^
NCTC 14809	*Salmonella enterica* subsp. *enterica* serotype Worthington	22	Obtained from a legacy strain archive	ERS222768^b^
NCTC 14810	*Salmonella enterica* subsp. *enterica* serotype Worthington	22	Obtained from a legacy strain archive	ERS222775^b^
NCTC 14811	*Salmonella enterica* subsp. *enterica* serotype Enteritidis	22	Obtained from a legacy strain archive	–
NCTC 14812	*Salmonella enterica* subsp. *enterica* serotype Hvittingfoss	22	Obtained from a legacy strain archive	–
NCTC 14813	*Salmonella enterica* subsp. *enterica* serotype Muenster	22	Obtained from a legacy strain archive	–
NCTC 14814	*Salmonella enterica* subsp. *enterica* serotype Oslo	22	Obtained from a legacy strain archive	–
NCTC 14815	*Salmonella enterica* subsp. *enterica* serotype Typhisuis	22	Obtained from a legacy strain archive	ERS222809^b^
NCTC 14816	*Salmonella enterica* subsp. *enterica* serotype Newport	22	Obtained from a legacy strain archive	ERS203131^b^
NCTC 14817	*Salmonella enterica* subsp. *enterica* serotype Thompson	22	Obtained from a legacy strain archive	ERS203129^b^
NCTC 14818	*Salmonella enterica* subsp. *enterica* serotype Typhisuis	22	Obtained from a legacy strain archive	ERS222823^b^
NCTC 14821	*Salmonella enterica* subsp. *enterica* serotype Gallinarum	22	Obtained from a legacy strain archive	ERS179744^b^
NCTC 14822	*Salmonella enterica* subsp. *enterica* serotype Moscow	22	Obtained from a legacy strain archive	–
NCTC 14823	*Salmonella enterica* subsp. *enterica* serotype Brandenburg	22	Obtained from a legacy strain archive	ERS203072^b^
NCTC 14824	*Salmonella enterica* subsp. *enterica* serotype Montevideo	22	Obtained from a legacy strain archive	ERS203086^b^
NCTC 14826	*Salmonella enterica* subsp. *enterica* serotype Aberdeen	22	Obtained from a legacy strain archive	ERS203133^b^
NCTC 14827	*Salmonella enterica* subsp. *enterica* serotype Gallinarum	22	Obtained from a legacy strain archive	ERS203130^b^
NCTC 14829	*Salmonella enterica* subsp. *enterica* serotype Aberdeen	22	Obtained from a legacy strain archive	ERS179724^b^
NCTC 14831	*Salmonella enterica* subsp. *enterica* serotype Enteritidis	22	Obtained from a legacy strain archive	ERS179709^b^
NCTC 14832	*Salmonella enterica* subsp. *enterica* serotype Javiana	22	Obtained from a legacy strain archive	ERS179671^b^
NCTC 14833	*Salmonella enterica* subsp. *enterica* serotype Newport	22	Obtained from a legacy strain archive	–
NCTC 14835	*Salmonella enterica* subsp. *enterica* serotype Typhimurium	22	Obtained from a legacy strain archive	–
NCTC 14842	*Salmonella enterica* subsp. *enterica* serotype Oranienburg	22	Obtained from a legacy strain archive	ERS179756^b^
NCTC 14843	*Salmonella enterica* subsp. *enterica* serotype Newport	22	Obtained from a legacy strain archive	–
NCTC 14844	*Salmonella enterica* subsp. *enterica* serotype Typhimurium	22	Obtained from a legacy strain archive	–
NCTC 14845	*Salmonella enterica* subsp. *enterica* serotype Typhimurium	22	Obtained from a legacy strain archive	–
NCTC 14846	*Salmonella enterica* subsp. *enterica* serotype Eastbourne	22	Obtained from a legacy strain archive	ERS179759^b^

The 19 type strains are denoted by T in the table above and throughout this manuscript. All strains are listed alongside their NCTC identifiers, species name, primary reference, attribute and whole-genome sequence data identifier where these data are available through accessible WGS databases. Seventy-three of 101 strains have available whole-genome sequence data accessible via the NCBI in at least one format. Whole-genome sequence data associated with the above strains are available in two formats: whole-genome sequence assemblies denoted with a or whole-genome sequence reads (Illumina) denoted with b. In each instance, the methods of generation and analysis of the whole-genome sequence data are detailed in each strain’s primary reference/associated citation.

Of the 101 bacterial strains, the majority (*n*=61) were obtained from a legacy strain archive: the Murray collection [22]. Ten are the type strains of novel taxa, 8 are type strains which have been previously described with validly published names, 2 strains are veterinary isolates, 2 are from human clinical sources, 15 are antimicrobial resistant strains and 3 are laboratory reference strains.

The full details for each strain (citations for the full taxon descriptions, strain provenance, validated growth conditions, typing information etc.) can be obtained from Table S1 and the NCTC online catalogue; https://www.culturecollections.org.uk/products/bacteria-and-mycoplasmas.

### Novel taxa made available in 2023

The ten type strains of novel taxa listed in Table 1 were all isolated from animals, except for NCTC 14661^T^ *Vibrio floridensis*. The strains were isolated in 2016 (*n*=1), 2018 (*n*=2), 2020 (*n*=5) and 2021 (*n*=2) and were isolated either in the UK or the USA.

NCTC 14661^T^ *V. floridensis* [13] was isolated from samples of a cyanobacterial bloom taken from the Indian River Lagoon in Florida, USA. This strain is described as phylogenetically closely related to the notable human pathogen *Vibrio vulnificus*. However, the two species differ in various aspects of their pathogenicity phenotype, i.e. NCTC 14661^T^ *V. floridensis* is demonstrated to be less resistant to normal pooled human serum, more readily phagocytized by THP-1 monocytes and less cytotoxic to THP-1 monocytes when compared to *V. vulnificus* strain CMCP6.

NCTC 14625^T^ *Terrisporobacter hibernicus* [12] is a spore-forming anaerobic species originally isolated from bovine faeces in Northern Ireland, UK, and NCTC 14451^T^ *Staphylococcus canis* [10] (of similar provenance to NCTC 14452^T^ *Staphylococcus caledonicus*) is a coagulase-negative species isolated from a multi-site swab of a healthy dog in Scotland, UK.

NCTC 14723^T^ *Staphylococcus marylandisciuri* and NCTC 14727^T^ *Streptococcus sciuri* were both isolated from the faeces of ‘apparently healthy’ Eastern Grey Squirrels (*Sciurus carolinensis*) in the USA [15]. NCTC 14721^T^ *Neisseria montereyensis* [14] was isolated from the oropharynx of a wild California sea lion (*Zalophus californianus*), also in the USA. The most closely related species prior to the description of *Neisseria montereyensis* was *Neisseria zalophi* [23, also isolated from the oral cavity of California sea lions.

NCTC 14562^T^ *Microbacterium pullorum*, NCTC 14700^T^ *Oerskovia rustica*, NCTC 14701^T^ *Clostridium gallinarum* and NCTC 14703^T^ *Sporosarcina quadrami* were isolated as a part of a single study into the microbiota of the chicken gut [11]. These strains were described in Gilroy *et al*. [11]; however, the names were not validly published at the time due to publication outside of the *IJSEM*. In 2024, the names became validly published in the *IJSEM* through publication of the names within the *IJSEM* [24].

In addition to these strains, NCTC 14574 ‘Candidatus Phocaeicola gallinarum’ was made available in 2023. The name of this strain is not validly published according to Oren and Göker [25] due to confusion surrounding the attachment of the name to genomic data and the original description. NCTC 14574 is categorized as a contemporary or significant veterinary isolate within Table 1.

The descriptions of the above validly named novel taxa, excluding NCTC 14574, were published in 2021 (*n*=1), 2023 (*n*=5) and 2024 (*n*=4).

### Existing taxa made available in 2023

Eight strains were made available from the NCTC in 2023 that are type strains of previously described taxa, with the descriptions published prior to accession. Seven were obtained via strain exchange with the Culture Collection of the University of Gothenburg (CCUG) (Göteborg, Sweden) and one from the Deutsche Sammlung von Mikroorganismen und Zellkulturen (DSMZ) (Braunschweig, Germany).

Previously, it was noted that four *Streptococcus* type strains belonging to previously described species were included in this group of strains. This trend continues with the accession of NCTC 14686^T^ *Streptococcus pluranimalium*, NCTC 14687^T^ *Streptococcus ictaluri* and NCTC 14688^T^ *Streptococcus plurextorum*, known pathogens of multiple mammalian species, Channel Catfish (*Ictalurus punctatus*), and pigs (*Sus domesticus*), respectively [19,26,28].

Two type strains belonging to the genus *Legionella* were also made available in 2023: NCTC 14628^T^ *Legionella drozanskii* and NCTC 14630^T^ *Legionella maioricensis* [16,17]. Including these two strains, the type strains of more than two-thirds of the 68 described *Legionella* species with validly published names are currently maintained within the NCTC at the time of writing.

NCTC 14681^T^ *Neisseria perflava* [29] was also added to the NCTC. In contrast to the prolific human pathogens within the genus *Neisseria* (e.g. *Neisseria gonorrhoeae* and *Neisseria meningitidis*), this species is frequently described as being a commensal *Neisseria* species in the scientific literature [18].

NCTC 14684^T^ *Dolosigranulum pigrum* [30] is the type strain of a species that was originally described in 1993, following the independent isolation of two ‘*Gemella*-like organisms’ in 1988 and 1991 from human clinical sources. While this species shows ‘some phenotypic similarity to gemellas’, the original 16S rRNA phylogeny published in 1993 supported the classification of this strain to a novel genus. Since the description of this species in 1993, it has been infrequently reported as being isolated from instances of human infection [31,32] and has also been described as a bacterium that has a beneficial effect on human health [33], which highlights this species’ complicated relationship with human health, other bacterial pathogens and other members of the human microbiome.

NCTC 14693^T^ *Lacrimispora algidixylanolytica* [20,34] is the type strain of an obligately anaerobic, psychrotolerant, xylan-degrading species. Of the eight type strains of previously described taxa with validly published names, only one was obtained from the DSMZ. It serves as a replacement for NCTC 13238^T^ *Lacrimispora algidixylanolytica*, the NCTC freeze-dried stocks of which had lost viability.

NCTC 14717 *Erysipelothrix rhusiopathiae* was isolated from a southern sea otter (*Enhydra lutris*) and represents the only non-type strain or proposed type strain originally isolated from an animal source cited in this manuscript.

NCTC 14851 and NCTC 14852 belong to the recently emerged and epidemiologically significant *Streptococcus pyogenes* lineage M1UK. Both strains were isolated from blood culture. The M1UK lineage belongs to the *emm*1 subtype of *Streptococcus pyogenes* and exhibits increased exotoxin A/erythrogenic toxin production (SpeA, a *Streptococcus pyogenes* virulence factor) [35] and is now reported to be the dominant source of invasive streptococcal infections in the UK [36].

Thirteen of the 15 antimicrobial-resistant (AMR) strains (NCTC 14894–NCTC 14898, NCTC 14900–NCTC 14902 and NCTC 14905–NCTC 14909) all belong to the Lancefield group B species *Streptococcus agalactiae*, which is a leading cause of neonatal sepsis. All 13 strains were isolated from within the UK. All strains were isolated from adult human blood, except NCTC 14909, which was isolated from pus following an instance of infection in a human. These strains specifically were examined in a study of group B *Streptococcus* (GBS), wherein antimicrobial gene content for each strain was determined and novel transposons and integrative conjugal elements identified [21]. This is within the context that ‘In the UK, GBS resistance to clindamycin and erythromycin has increased from 3% in 1991 to 11.9% (clindamycin) and 20.2% (erythromycin),’ as reported by the same study.

Two further AMR strains both belong to the species *Leclercia adecarboxylata*. NCTC 14653 *Leclercia adecarboxylata* carries a blaKPC carbapenemase gene, and NCTC 14660 *Leclercia adecarboxylata* carries a *bla*_*OXA-48*_-like gene. NCTC 14653 has been determined to be phenotypically resistant to amoxicillin/clavulanic acid, ampicillin, cefotaxime, ertapenem and piperacillin/tazobactam.

The three strains categorized as laboratory reference strains are NCTC 14916, NCTC 14692 and NCTC 14679. NCTC 14916 *Escherichia coli* is a sub-strain of *Escherichia coli* K12: K12 DH5. The K12 DH5 sub-strain does not contain the F plasmid, in contrast to some *Escherichia coli* K12 strains such as NCTC 10538, which do. NCTC 14692 *Escherichia coli* is the MC1061 strain, also a K12 derivative strain that does not contain an F plasmid, which is frequently used as an efficient transformation vector. NCTC 14679 *Clostridium perfringens* has been used for many years as a quality control strain in testing the integrity of anaerobic atmospheres [37]. Recently, it has been cited as a quality control strain in European Committee on Antimicrobial Susceptibility Testing (EUCAST) guidelines for antimicrobial susceptibility testing (AST), as the degree of anaerobicity of the incubation atmosphere can affect the result of AST. NCTC 14679 is susceptible to metronidazole and, unusually, is reportedly mildly aerotolerant.

61 strains were obtained and made available from the Murray collection of pre-antibiotic era *Enterobacteriaceae*, the nature of which has been discussed previously [22]. Fifty-seven non-Typhi and non-Paratyphi *Salmonella* strains predominate this group of strains of 31 different serotypes. The remaining strains (NCTC 14744, NCTC 14745, NCTC 14746 and NCTC 14805) are all strains of *Escherichia coli*. All *Escherichia coli* strains are negative for Shiga toxin genes and belong to non-O157 serotypes. Interestingly, 33 of the 61 strains’ non-Murray identifiers as reported by Baker *et al.* [22] are NCTC strain identifiers, which suggests that Prof. Everitt George Dunne Murray (who collected the strains between 1917 and 1954) appeared to have acquired strains from the NCTC. Of the NCTC identifiers represented in the Murray collection metadata, only three remain represented in the NCTC today (NCTC 3378, NCTC 4444 and NCTC 4785). Strains listed under the other NCTC identifiers have been discarded from the NCTC in the intervening 71 years.

### Overview of bacterial strains made available by the NCTC between 2017 and 2023

**Fig. 1.**
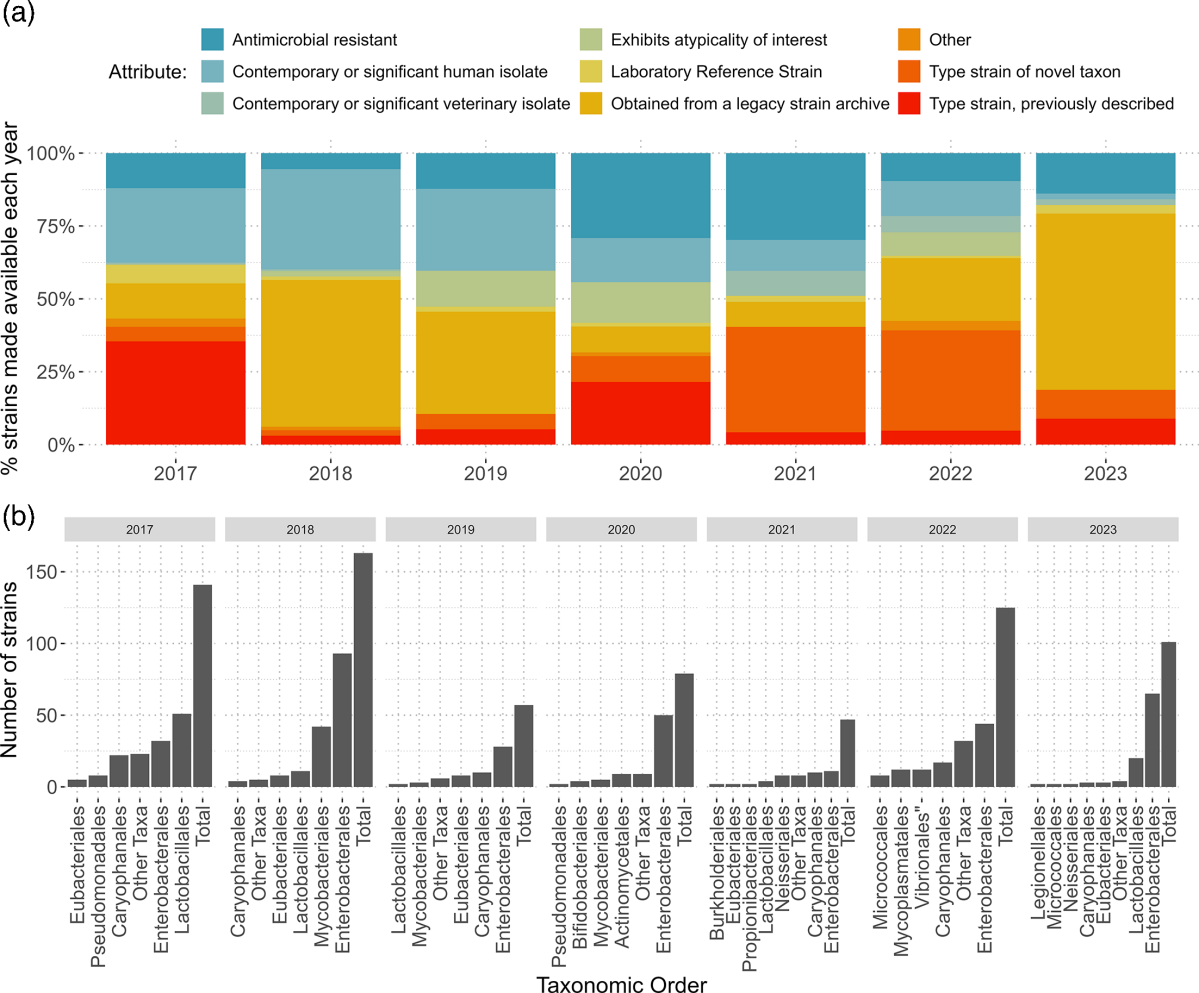
The proportion of strains made available each year subdivided by strain attribute. The number of strains made available each year for 2017, 2018, 2019, 2020, 2021, 2022, and 2023 is, respectively, 141, 163, 58, 79, 47, 125 and 101, totalling 714 strains.

Fig. 1 illustrates the proportion of strains made available each year from and including 2017 to 2023 as assigned to the described categories (a) and the number of strains made available each year from each taxonomic order (b).

The total number of strains made available in any given year fluctuates from 47, in 2021, to 163, in 2019. The mean and median average number of strains made available in any given year between 2017 and 2023 are 102 and 101, respectively.

Strains from 63 taxonomic families belonging to 36 different taxonomic orders were made available from the NCTC between 2017 and 2023. Of all microbial taxa, strains from *Enterobacterales* were most numerously made available each year except for 2017, when more bacterial strains belonging to the order *Lactobacillales* were made available.

### Commentary on trends in strains made available between 2017 and 2023

Strains obtained from legacy strain archives are the only group of strains that have made up more than half of the number of strains made available in any given year, in 2018 and 2023. Type strains, whether those from novel taxa or type strains of taxa that were previously described prior to accession, on average make up around 25% of strains made available within a given year. Between 2017 and 2022, the proportion of strains made available that were accessioned as proposed type strains of novel taxa steadily increased.

AMR strains and strains that are considered contemporary or significant human clinical isolates are consistently well represented each year; collectively, these strains comprise ~33% of the strains made available each year. Strains that are considered laboratory reference strains are made available each year in small numbers. Strains of veterinary provenance are accessioned and made available in lower numbers and more intermittently than those of human provenance, as are strains with atypicality of interest, although in greater numbers than strains of veterinary interest.

Taxonomically, strains made available from the NCTC are most frequently *Enterobacterales*, with *Lactobacteriales* being the second most frequent.

### Concluding remarks

In line with a determined average of 101/102 distinct bacterial strains accessioned per year, the 101 strains made available to the scientific community in 2023 represent an ordinary growth of the number of bacterial strains available from the NCTC. The current size of the NCTC is around 6,000 bacterial strains, with more than 700 of these (>10 %) being made available between 2017 and 2023.

In examining wider trends, provision of strains of veterinary provenance is intermittent and the number and diversity of these bacterial strains that are made available by the collection could stand to be improved. There is also over-representation of strains obtained from legacy strain archives in the 2023 strain cohort, contrasting with previous years, where contemporaneous strains are more abundant. While reclaiming older bacterial strains from being lost to science is a valuable endeavour due to their status as irreplaceable biological materials, this activity must be matched by the provision of more ‘contemporary’ strains by the NCTC.

A depth and diversity of AMR *Streptococcus agalactiae* strains have been made available in 2023 (where previously there was a paucity of AMR *Streptococcus agalactiae* of any kind held by the collection), alongside two strains of a *Streptococcus pyogenes* lineage that now predominates in the UK, highlighting the more contemporary members of the 2023 strain cohort. Accessioning strains with AMR phenotypes that carry diverse AMR determinants allows the NCTC to provide a greater range of reference material globally that can be used (for example) in the development of new antimicrobials or as references in microbial testing. Commensurately, making more strains that belong to emergent bacterial lineages available to the scientific research community allows for infectious disease research on strains that are highly representative of the current infectious disease burden and enhances the NCTC’s utility as a public health resource.

Numerous type strains of novel taxa were made available in 2023, and similarly to the 2022 strain cohort, this included strains with similarities or close phylogenetic relationships to established bacterial pathogens. Increasing the number of type strains (and their associated genomic data) that are available from the NCTC strengthens the collection’s facility as a taxonomic resource to scientists globally. While microbial taxonomy is increasingly driven by genomic analysis, access to a range of type strains allows for more robust and reproducible comparative analysis between microbial taxa. Making available a significant number of type strains each year, both those that were proposed as type strains at the point of accession and those with already validly published names, showcases the NCTC’s active function as a type culture collection with active engagement with prokaryotic taxonomists.

It has been previously discussed that the number and diversity of bacterial strains made available in the year 2022 were a product of mutualistic collaboration with the base of scientists, which the NCTC services [2]. This remains true for 2023.

More information on depositing bacterial strains into the NCTC, a free service, is available on the NCTC/UKHSA Culture Collections website:https://www.culturecollections.org.uk.

## The NCTC 2023 Depositors Cohort

The NCTC 2023 Depositors Cohort consists of individuals who deposited strains into the NCTC and those instrumental in preparing the strains for submission to the NCTC.

The NCTC 2023 Depositors Cohort are Dr Vicki Chalker (UK Health Security Agency, Colindale, London, UK), Dr Katie Hopkins (Antimicrobial Resistance and Mechanisms Service, Antimicrobial Resistance and Healthcare Associated Infections Unit, UK Health Security Agency, Colindale, London, UK), Prof. Ulrik S. Justesen (Department of Clinical Microbiology, Odense University Hospital, Denmark), Molly Mitchell (University College Dublin, Centre for Food Safety, School of Public Health, Physiotherapy and Sports Science, Dublin, Ireland), Prof. Robert George Everitt Murray, Prof. Mark Pallen (Quadram Institute, Norwich, UK), Dr Gavin Paterson (Royal (Dick) School of Veterinary Studies, University of Edinburgh, Edinburgh, Scotland, UK) and Prof. Owen B. Spiller (School of Medicine, Cardiff University, Cardiff, Wales).

Strains were obtained from the DSMZ (Braunschweig, Germany) and CCUG (Gothenburg, Sweden) as part of strain exchange programmes.

## Supplementary material

10.1099/acmi.0.001015.v3 Uncited Fig. S1.

10.1099/acmi.0.001015.v3 Uncited Supplementary Material 1.

## Abbreviations

AMR: antimicrobial resistant
AST: antimicrobial susceptibility testing
CCUG: Culture Collection of the University of Gothenburg
DSMZ: Deutsche Sammlung von Mikroorganismen und Zellkulturen
EUCAST: European Committee on Antimicrobial Susceptibility Testing
GBS: group B *Streptococcus*
ICNP: International Code of Nomenclature of Prokaryotes
*IJSEM*: *International Journal of Systematic and Evolutionary Microbiology*
NCTC: National Collection of Type Cultures
UKHSA: UK Health Security Agency
WGS: whole-genome sequencing
