# Supplementary figures and images for: Notification of bacterial strains made available by the UK National Collection of Type Cultures in 2023
Source: Access Microbiol. 2025 Oct 22;7(10):001015.v3. doi: 10.1099/acmi.0.001015.v3 (PMC12543791; doi:10.1099/acmi.0.001015.v3)

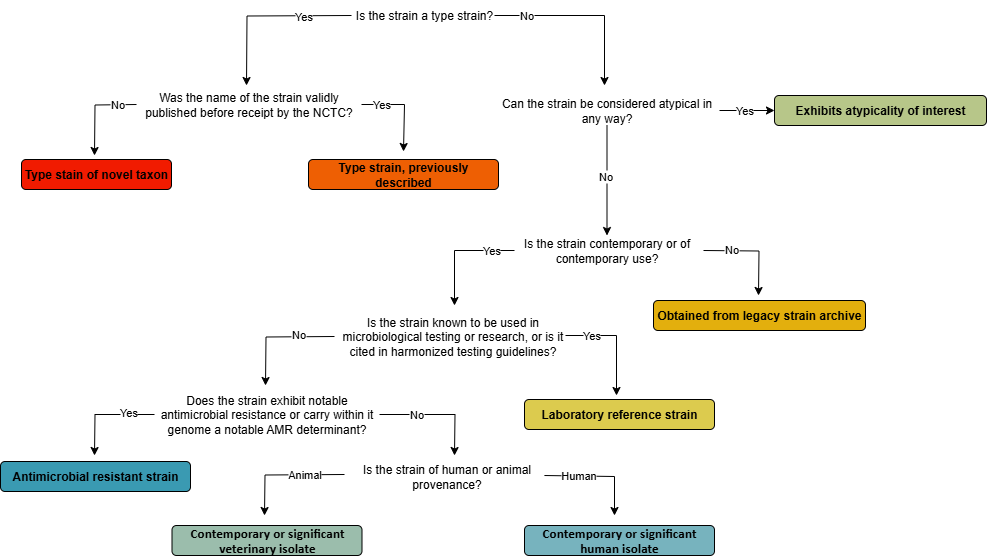

Supplement: Uncited Fig. S1. [file acmi-7-01015-s001.png]
